# Overexpression of Heat Shock Protein 72 Attenuates NF-κB Activation Using a Combination of Regulatory Mechanisms in Microglia

**DOI:** 10.1371/journal.pcbi.1003471

**Published:** 2014-02-06

**Authors:** Patrick W. Sheppard, Xiaoyun Sun, Mustafa Khammash, Rona G. Giffard

**Affiliations:** 1 Department of Mechanical Engineering, University of California, Santa Barbara, Santa Barbara, California, United States of America; 2 Department of Biosystems Science and Engineering, ETH-Zurich, Basel, Switzerland; 3 Department of Anesthesia, Stanford University, Stanford, California, United States of America; Johns Hopkins University, United States of America

## Abstract

Overexpression of the inducible heat shock protein 70, Hsp72, has broadly cytoprotective effects and improves outcome following stroke. A full understanding of how Hsp72 protects cells against injury is elusive, though several distinct mechanisms are implicated. One mechanism is its anti-inflammatory effects. We study the effects of Hsp72 overexpression on activation of the transcription factor NF-κB in microglia combining experimentation and mathematical modeling, using TNFα to stimulate a microglial cell line stably overexpressing Hsp72. We find that Hsp72 overexpression reduces the amount of NF-κB DNA binding activity, activity of the upstream kinase IKK, and amount of IκBα inhibitor phosphorylated following TNFα application. Simulations evaluating several proposed mechanisms suggest that inhibition of IKK activation is an essential component of its regulatory activities. Unexpectedly we find that Hsp72 overexpression reduces the initial amount of the RelA/p65 NF-κB subunit in cells, contributing to the attenuated response. Neither mechanism in isolation, however, is sufficient to attenuate the response, providing evidence that Hsp72 relies upon multiple mechanisms to attenuate NF-κB activation. An additional observation from our study is that the induced expression of IκBα is altered significantly in Hsp72 expressing cells. While the mechanism responsible for this observation is not known, it points to yet another means by which Hsp72 may alter the NF-κB response. This study illustrates the multi-faceted nature of Hsp72 regulation of NF-κB activation in microglia and offers further clues to a novel mechanism by which Hsp72 may protect cells against injury.

## Author Summary

Inducing heat shock or overexpressing certain heat shock proteins (HSPs) is known to protect against brain injury, such as that resulting from stroke. Understanding the mechanisms underlying protection at the cellular and molecular level is a subject of intense research, as such knowledge may prove beneficial in designing future therapies. Regulation of the activation of the key inflammatory transcription factor Nuclear Factor κB (NF-κB) is believed to be one critical mechanism. However how its activation is altered by Hsp72 remains unresolved. Here we examine NF-κB signaling in microglia cells overexpressing Hsp72, combining experimentation and mathematical modeling. We show that Hsp72 affects signaling using at least two essential and distinct mechanisms: attenuation of upstream kinase (IKK) activity and reduction of steady state NF-κB protein levels. We provide numerical evidence suggesting that neither mechanism in isolation is sufficient to account for the observed signaling. Furthermore, our observations suggest an intriguing additional level of regulation of gene expression and protein synthesis of the IκBα inhibitor, which opens interesting new avenues of research. These results provide novel insight into the mechanisms by which Hsp72 may regulate inflammation and protect brain cells from injury.

## Introduction

Hsp72 is the major cytosolic inducible form of the 70 kDa family of heat shock proteins (HSP70). Overexpression of Hsp72 is known to protect cells from injury and is positively associated with outcome in models of stroke [1], [2], [3], [4], [5]. Besides the role it plays as a molecular chaperone, Hsp72 is also an important mediator in intracellular signaling including inflammatory and cell death signaling [6]. One of the important mechanisms by which Hsp72 affects cellular outcomes is its regulation of the proinflammatory transcription factor Nuclear Factor κB (NF-κB) [7]. Activation of microglia following stroke with production of numerous signaling and immune modulatory proteins downstream of NF-κB make microglia important potential targets for therapeutic intervention [8], [9]. NF-κB activation in microglia is attenuated when cells overexpress Hsp72 [6], [10], suggesting that Hsp72 attenuation of NF-κB activation may be a key contributor to cytoprotection.

NF-κB is a family of dimeric transcription factors that regulate the transcription of hundreds of genes in a coordinated manner in response to an inducing signal. In resting cells NF-κB is found primarily in the cytosol bound to its inhibitor IκB proteins. Upon stimulation by cytokines or other inducers, IκB proteins are targeted for proteasomal degradation by the IκB kinase (IKK). Once IκB is degraded, NF-κB translocates to the nucleus to activate gene expression. Among its target genes are its own inhibitors and other regulatory proteins that form a complex network that tightly regulates the dynamic response and gene transcription [11]. Expression of the IκBα and IκBε inhibitors is strongly induced to provide direct negative feedback of NF-κB [12]. Another early target, A20, attenuates activation of inhibitor of IκB kinase (IKK) and provides an additional layer of negative feedback [13].

Which mechanism or mechanisms Hsp72 uses to regulate NF-κB in microglia is unclear. In protein binding studies from Hsp72-transgenic mice and mixed cultures of glial cells overexpressing Hsp72, attenuation of NF-κB activation was shown to be dependent on association between Hsp72 and NF-κB and IκBα, but not IKKγ/NEMO [14]. In contrast a study in a different cell type found that Hsp72 associates directly with the IKKγ/NEMO subunit of the IKK complex but not with the IκBα:NF-κB complex [15]. Interactions between Hsp72 and factors further upstream of IKK have also been identified [16]. To add to the confusion others have observed that heat shock can prevent IκBα degradation without affecting its phosphorylation [17], or that Hsp72 interacts at both the level of IKK activation and with the IκBα:NF-κB complex [18], [19]. Given the complex nature of NF-κB signaling and the many possible sites of regulation, better understanding how Hsp72 regulates signaling is vital and will contribute to our ability to design therapeutic strategies that target this pathway.

Here we examine Hsp72 regulation of NF-κB activation in microglial cells subjected to the inflammatory cytokine tumor necrosis factor-α (TNFα). Using transfected cell lines stably overexpressing Hsp72 we observe that Hsp72 attenuates NF-κB signaling compared to controls. We then utilize a mathematical model to simulate several potential regulatory effects of Hsp72 overexpression on NF-κB activation. These effects are implemented in the model as constant changes to reaction rate parameters that are assumed to be altered constitutively as a result of Hsp72 overexpression. An inconsistency between the model and the data led us to observe a novel effect: in Hsp72 overexpressing cells the p65/RelA subunit of NF-κB is present at lower levels. Using the model we show that reduced p65 is able to account for the observed reduction in total IκBα observed in unstimulated cells but does not fully account for the attenuated IKK activation. This suggests that Hsp72 must act both at the level of inhibiting IKK activation and by altering steady state protein levels of NF-κB. Additional investigation reveals that IκBα mRNA is induced at higher levels in Hsp72 expressing cells following TNFα stimulation.

## Results

### Overexpression of Hsp72 reduces IKK kinase activity and NF-κB DNA binding activity in microglia BV2 cells

**Figure 1:**
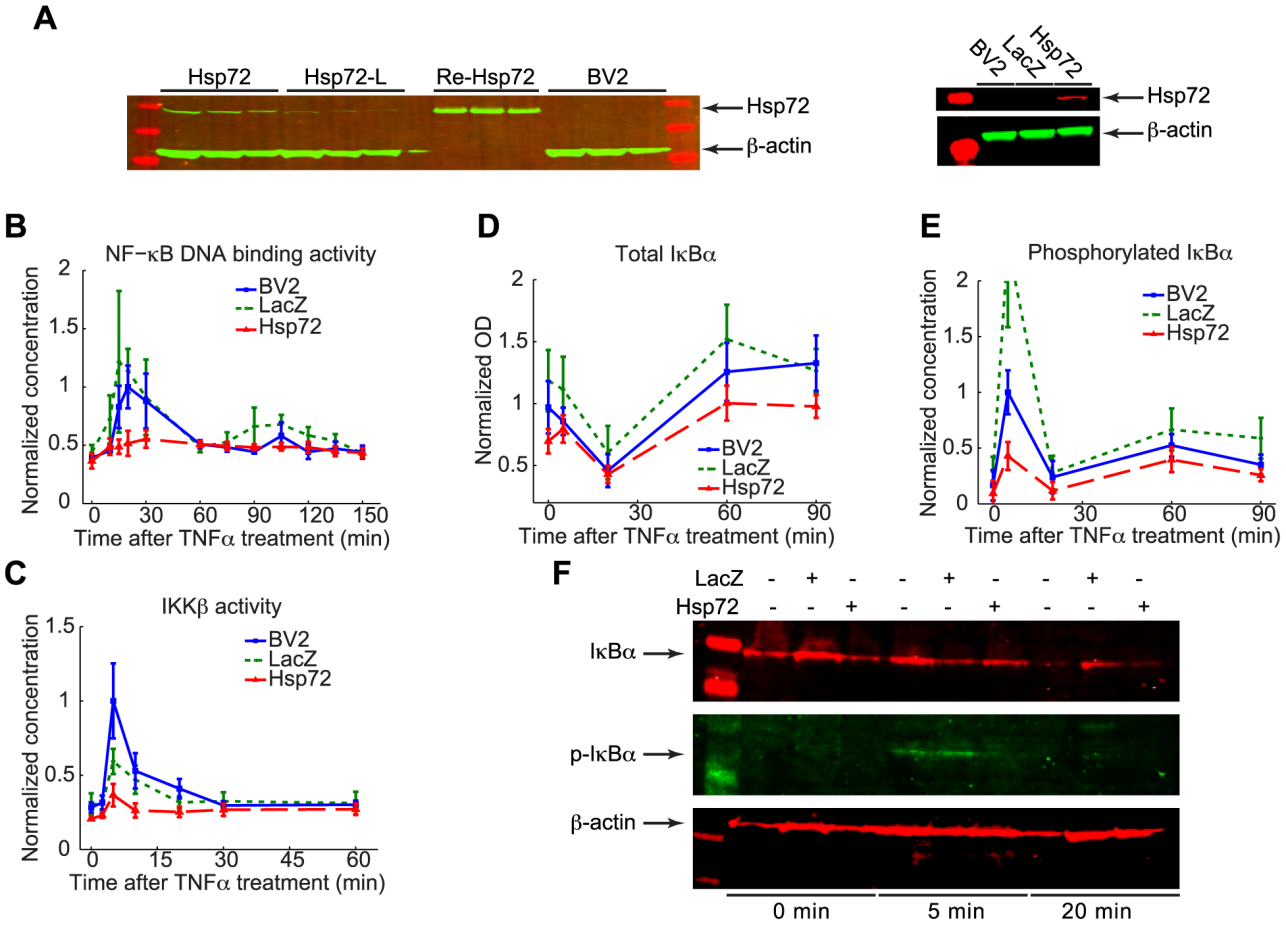
NF-κB signaling in response to TNFα stimulus is attenuated in BV2 cells overexpressing Hsp72 cells. A. Western blot assays of BV2 cells stably overexpressing Hsp72 at high levels (Hsp72) and low levels (Hsp72-L) compared with recombinant Hsp72 (Re-Hsp72), control cells (BV2), and control cells transfected with LacZ vector (LacZ). B. NF-κB p65/RelA DNA binding activity is significantly reduced in Hsp72 cells at 15, 20, 30, 105 min time points compared to other cell types. Concentrations were measured with ELISA and normalized with respect to the average measured p65 activity in BV2 cells at 20 min (n = 4 at 150 min for LacZ, n = 6 for all other time points and cell types). C. IKKβ kinase activity following treatment with 10 ng/ml TNFα at 0 min is significantly lower in Hsp72 cells at 0, 2.5, 5, 10 and 20 min. Concentrations were measured with ELISA and normalized with respect to average IKK activation in BV2 cells at 5 min (n = 6 at each time point). D. Total IκBα protein is reduced in Hsp72 cells compared to other cell types at 0, 60 and 90 min. Total IκBα levels were measured using ELISA optical density (OD) readings and normalized with respect to the average OD in BV2 cells at 0 min (n = 9 at each time point). E. Ser-32 phosphorylated IκBα is lower in Hsp72 cells than other cell types at 5, 20, 60 and 90 min. Concentrations were measured with ELISA and normalized with respect to the average level in BV2 cells at 5 min (n = 9 at each time point). F. Western blot assays of total and Ser-32 phosphorylated IκBα protein from whole cell extracts.

To study the effects of Hsp72 overexpression in microglia, the mouse microglial cell line BV2 was transfected with a retroviral vector to generate cell populations stably overexpressing the inducible Hsp72 protein in the absence of heat shock. Two cell lines expressing Hsp72 at different levels were isolated. Western blot assays verified that the transfected BV2/Hsp72 cell lines expressed Hsp72 protein under resting conditions, whereas BV2 control cells and LacZ transfection control cells contained no measurable Hsp72 (Figure 1A).

Cells were treated with 10 ng/ml TNFα to induce activation of the canonical NF-κB pathway. The time course of NF-κB activity in each cell population was assessed for 150 min following TNFα addition to the medium by measuring DNA binding activity of the p65/RelA NF-κB subunit. Cells expressing Hsp72 at high levels (Hsp72) exhibited similar NF-κB activity in the absence of stimulus, but the amount of NF-κB activation in response to TNFα was reduced to <50% of that seen in control BV2 cells (Figure 1B). Reduced nuclear translocation of p65 in response to TNFα in Hsp72 cells was confirmed using immunostaining (Figure S1). Kinase activity of the IκB kinase (IKK) was also assessed following TNF addition. IKK activation was significantly reduced but not altogether abolished in Hsp72 cells compared to controls (Figure 1C). Cells expressing Hsp72 at lower levels (Hsp72-L) exhibited no significant differences in NF-κB or IKK activation compared to controls (unpublished data), suggesting that attenuation of NF-κB signaling requires a sufficient quantity of Hsp72 protein in the cell. Only high Hsp72-expressing cells were used for subsequent analysis.

Levels of total IκBα protein and Ser32-phosphorylated IκBα (p-IκBα) were additionally measured at several times following TNFα treatment. All cell types showed similar qualitative behavior, with total protein levels significantly decreased 20 min following stimulus before overshooting to higher levels at 60 min and 90 min (Figure 1D). However levels of total IκBα were lower initially and at later time points in Hsp72 cells than in control cells. Phosphorylation of IκBα was also reduced substantially in Hsp72 cells compared to controls (Figure 1E). The reductions in total and phosphorylated IκBα observed in Hsp72 cells were confirmed using Western Blot (Figure 1F).

### Computational model suggests inhibition of IKK activity by Hsp72 is essential

Hsp72 has been demonstrated previously to regulate NF-κB activation in multiple cell types subjected to different stimuli depending on the context. The reported Hsp72 regulatory mechanisms roughly fall into two categories. In the first, Hsp72 interacts with downstream members of the IκBα:NF-κB complex to prevent IκBα proteasomal degradation and subsequent NF-κB nuclear translocation. The second category includes Hsp72 interactions with upstream signaling components to inhibit activation of the IKK complex.

In order to examine which of the Hsp72 mechanisms are likely to be present in microglia, we first employed mathematical modeling to assess which possible mechanisms are consistent with the experimental data. Mathematical models are valuable tools for studying biological networks: inconsistencies between experimental observations and simulations of models constructed from known biological interactions can help identify missing essential pieces or guide investigation into previously unknown interactions [20]. A mathematical model describing the dynamic NF-κB response to TNFα in the microglial cell line BV2 was recently developed [21]. This model assumes that basal IKK activity is negligible. While the assays suggest a possible significant level of basal IKK activity (Figure 1 C), this assumption is justified from our other observation that initial concentrations of phosphorylated IκBα are nearly undetectable (Figure 1 E–F) coupled with earlier reports in fibroblasts that IKK activity does not contribute to basal turnover of IκBα protein [22].

**Figure 2:**
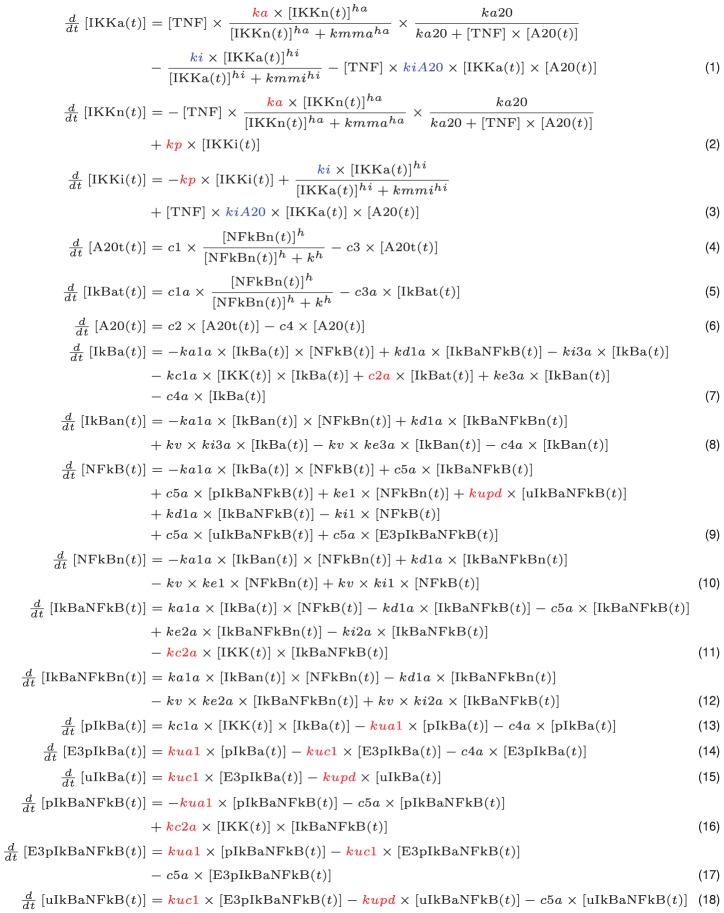
Ordinary differential equations describing NF-κB signaling network. Protein concentrations are denoted with brackets. Parameters altered to simulate reported and hypothetical interactions due to Hsp72 are colored red to denote inhibition and blue to denote enhancement. See also Tables 1 and 2 for biological interpretations of parameter modifications, and Supporting Information Tables S1 and S2 for parameter values and initial conditions used in the model.

We therefore adopted this model with slight modifications to analyze proposed Hsp72 regulatory mechanisms computationally. The system of ordinary differential equations (ODE) describing the model dynamics is shown in Figure 2.

We used this model to simulate the possible Hsp72 interactions by first making several key assumptions. From our initial experiments, Hsp72 was only observed to have a significant effect on NF-κB signaling when overexpressed at high levels. Because Hsp72 is not a known NF-κB gene target and is only expressed in resting transfected cells under the control of a constitutive promoter, we therefore assume that its concentration remains constant at a sufficiently high level throughout the entire time course considered during our experiments. Accordingly, Hsp72 is not explicitly included in the model as a reacting species; instead we assume throughout the paper that effects of constant Hsp72 overexpression are interpreted in the model as constant changes to specific reaction rates depending on where Hsp72 supposedly interacts. First we considered the effects of Hsp72 on upstream signaling by altering model parameters as summarized in Table 1 and explained below.

**Table 1:** Modifications to model parameters to simulate upstream and downstream regulatory interactions in the NF-κB signaling network.

Upstream parameters		
Change to model	Biological interpretation	Simulated results
Decreased *ka* (Equations 1–2)	Hsp72 inhibition of IKK activation through direct binding to NEMO [15], [18], [23]	Figure 3A, Figure S2A
	Hsp72 inhibition of IKK activation due to interactions with upstream components such as TRAF2 [16]or TRAF 6 [19]	Figure 3A, Figure S2A
Decreased *kp* (Equations 2–3)	Decreased recovery of inactivated IKK to neutral state (hypothetical)	Figure S2B
Increased *ki* (Equations 1, 3)	Increased auto-phosphorylation inhibition (hypothetical)	Figure S2C
Increased *kiA20* (Equations 1, 3)	Increase inhibition due to A20-like effects (hypothetical)	Figure S2D

Downstream interactions		
Change to model	Biological interpretation	Simulated results
Decreased *kc2a* (Equations 11, 16)	Hsp72 inhibition of IκBα phosphorylation [14]	Figure 3B, Figure S3A
Decreased *kupd* (Equations 9, 15, 18)	Hsp72 inhibition of IκBα proteasomal degradation [15], [18]	Figure S3B
Decreased *kua1* (Equations 13–14, 16–17)	Hsp72 mediated stabilization of phosphorylated IκBα protein [18]	Figure S3C
Decreased *kuc1* (Equations 14–15, 17–18)	Hsp72 mediated stabilization of phosphorylated IκBα complex [18]	Figure S3D

Hsp72 interactions with the NF-κB signaling network are interpreted as constant perturbations to rates corresponding to the relevant biological reaction. Equation numbers reference the complete model equations shown in Figure 2, with altered parameters colored in red (inhibition) or blue (enhancement).

**Figure 3:**
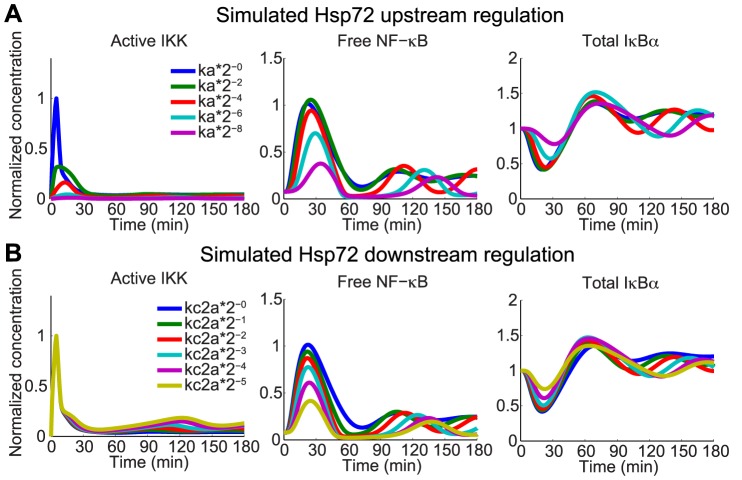
Modeling suggests Hsp72 attenuation of IKK is essential for differential IKK and NF-κB activation. A. Model simulations show expected dynamics of IKK, NF-κB and total IκBα protein when Hsp72 inhibits TNF-induced IKK activation. Each curve corresponds to simulations with rate *ka* multiplied by the factor indicated. B. Model simulations when Hsp72 is assumed to inhibit phosphorylation of IκBα (rate *kc2a*). When phosphorylation is sufficiently attenuated, peak NF-κB activation drops similar to levels observed experimentally, but IKK activation at early times is largely unaffected.

Hsp72 has been observed in some studies to inhibit IKK activation by directly binding NEMO within the IKK complex [15], [18], [23], or else by impairing IKK activation by interaction with yet further upstream components including TRAF2 [16]. We tested this mode of regulation in computer simulations by reducing the rates responsible for activation of IKK, and alternatively by enhancing inactivation of active IKK (Figures 3A and S2; Table 1). In particular, it was assumed that these upstream interactions effectively reduce the rate at which IKK is activated (model species [IKKa(t)], Figure 2), for which one interpretation in the model is a reduction of the activation rate, *ka*. Simulations showed that significant reduction of *ka* dramatically attenuated IKK activation and consequently reduced the amount of NF-κB activated and IκBα degraded (Figure 3A). In addition to these reported mechanisms, several other scenarios by which Hsp72 could affect upstream signaling are hypothetically possible and were explored using the model. Decreasing the rate of recovery of IKK from an inactivated state to a neutral state capable of further activation (rate *kp*, Figure 2) had minimal effect on reducing peak IKK, and only altered later dynamics of NF-κB (Figure S2B). In contrast, enhancing the rate at which active IKK becomes inactivated reduced peak IKK activation significantly (Figure S2C and S2D). However increasing the rate of auto-inhibition (rate *ki*, Figure S2C) still allowed a high amplitude first peak of NF-κB, potentially suggesting that it is an unlikely candidate for effective attenuation of NF-κB activation. Also of note was that reduction in only the activation rate delayed the peak of IKK activity once IKK activity was sufficiently reduced. This observation, however, may be a consequence of limitations of the IKK model given the lack of detailed experimental observation needed for more rigorous validation and do not necessarily exclude it as a plausible mechanism.

Yet other studies of Hsp72 overexpression found that Hsp72 instead stabilizes IκBα protein by directly binding to the NF-κB∶IκBα complex but not to IKK [14], or possibly by binding intermediate complexes possibly including IKK [18]. Accordingly alternative downstream scenarios were tested with the model by altering the reaction rates for steps involved in the degradation of IκBα and examining the simulated effects. The *in silico* results indicate that when Hsp72 interaction at this point is assumed to effectively decrease the phosphorylation rate, *kc2a*, by a factor greater than 10 fold, the amplitude of NF-κB activation is significantly reduced (Figure 3B). Such a mechanism could be the result of Hsp72 physically binding to the IκBα:NF-κB complex to inhibit phosphorylation by the IKK complex [14]. Irrespective of the strength of inhibition, however, IKK activation is initiated in a similar manner as in simulations at the nominal parameters, showing only discrepancies at later time points when NF-κB target gene expression is differentially regulated. Similar results were observed when IκBα phosphorylation was uninhibited but later steps needed for proteasomal degradation were instead assumed to be inhibited (implemented by reducing rates *kua1*, *kuc1*, or *kupd* in the model), as suggested elsewhere in the literature [17], [18] (Figure S3). While simulations suggest little change in terms of the responses of IKK, NF-κB, and total IκBα between inhibiting the phosphorylation step or steps further downstream, there was a considerably higher proportion of total IκBα found in a phosphorylated or ubiquitinated state when phosphorylation was allowed to occur. Such qualitative behavior observed in simulation would support a claim that such mechanisms are less likely to be the predominant means by which Hsp72 acts downstream.

Taken together with the experimental results, model simulations indicate that Hsp72 must act upstream of the NF-κB∶IκB complex in order to inhibit IKK activation following TNFα stimulus unless some unknown mechanism unaccounted for in the structure of the model is present, while no conclusion can yet be drawn regarding downstream regulation. One feature of the data wholly unaccounted for by simulations, however, was the reduction in total IκBα protein observed in Hsp72 cells (Figure 1C). We followed this up by assessing p65 levels.

### Hsp72 expression reduces steady state protein levels of IκBα and p65 NF-κB

**Figure 4:**
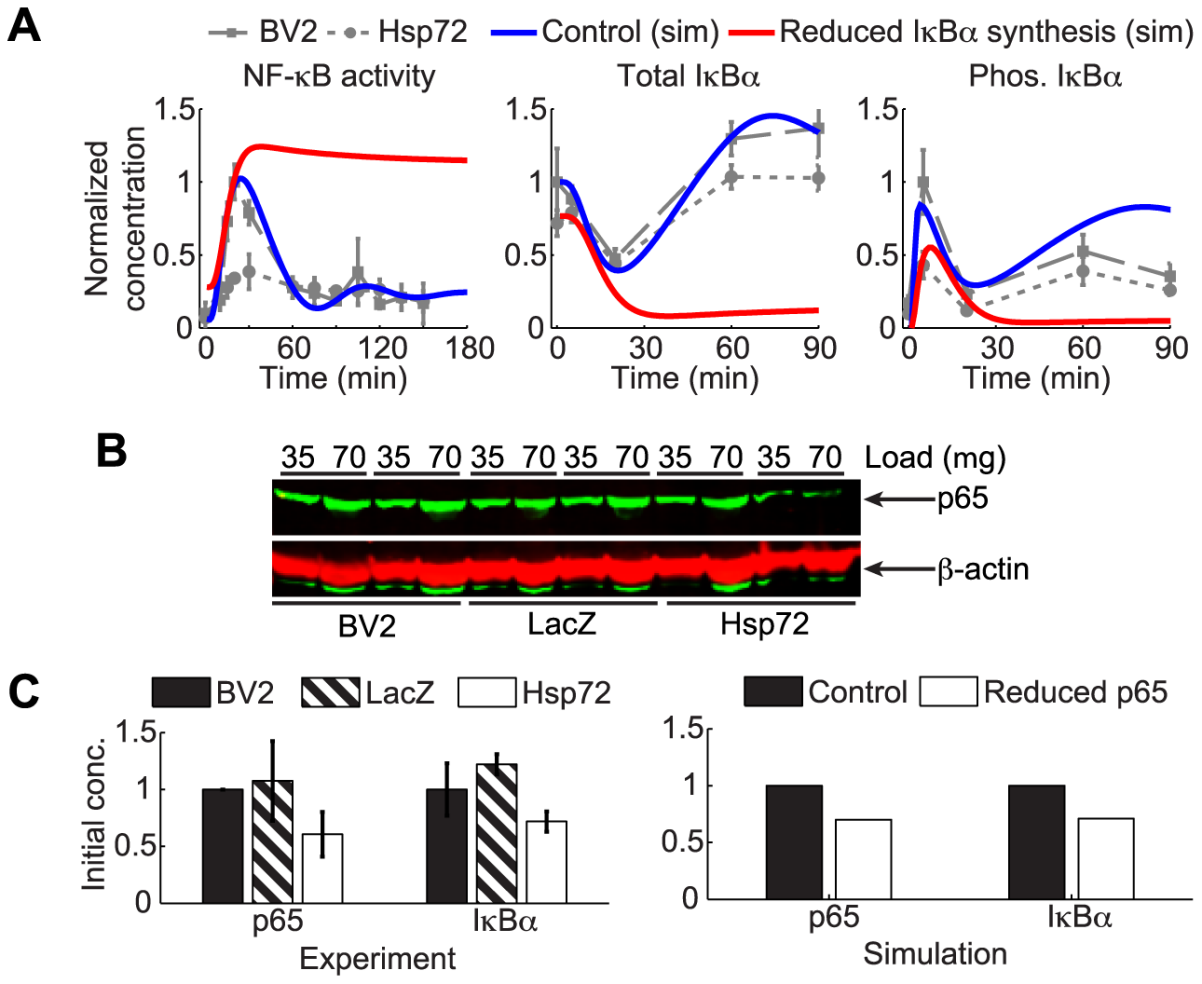
Hsp72 overexpression decreases total NF-κB-p65 in resting cells and reduces steady state IκBα protein levels. A. Model simulations directly reducing IκBα protein synthesis suggest mechanism inconsistent with data. Simulations were obtained from the model with nominal parameters (control, blue lines) and from the model with the rate of IκBα proteins translation (*c2a*) reduced to 1/30 the nominal level (red). Gray data points are rescaled from Fig. 1B. B. Representative western blot assay of p65 NF-κB in BV2, LacZ, and Hsp72 cells show that p65 levels are decreased in Hsp72 overexpressing cells compared to controls. C. Quantification of p65 western blot assays from multiple experiments compared with initial total IκBα protein levels as assessed by ELISA assay in Fig. 1F are shown (left panel) and compared with simulated initial concentrations (right panel). Data points for p65 are the averages from n = 5 independent measurements, each normalized with respect to the initial amount of p65 in untreated BV2 cells. IκBα data points are averages from (n = 9) independent measurements and normalized with respect to the mean initial measurement in untreated BV2 cells. Mathematical model simulated with 70% initial total NF-κB correctly accounts for the reduction in steady state IκBα levels observed from experiments. The mean protein levels in Hsp72 cells are significantly different (P<0.05) from BV2 cells as determined by t-test (p65) and different from both BV2 and LacZ cells as determined by ANOVA and Newman-Keuls *post hoc* test (IκBα).

A reduction in total IκBα levels could conceivably be achieved by negative regulation of its basal synthesis by some yet to be determined mechanism. To test whether such a mechanism is likely to occur, we simulated the model assuming that Hsp72 interactions alter several model parameters (Table 2). First we simulated inhibition of IκBα protein synthesis by decreasing rate *c2a* of the model (see Eq. (7) in Figure 2) until basal IκBα was reduced to a level consistent with experiments. Simulations show that such a reduction in IκBα protein synthesis rate causes a precipitous drop in total IκBα concentration following stimulation together with elevated basal and sustained transient NF-κB activation (Figure 4A). Interpreted in the cellular context, the simulations suggest that direct inhibition of IκBα in the network would severely impair the ability of the cell to synthesize adequate *de novo* IκBα protein to terminate NF-κB activation following stimulus This would imply that such a regulatory mechanism is unlikely to reduce initial IκBα, leading us to examine alternative mechanisms.

**Table 2:** Modified model parameters to simulate possible regulation of steady state protein levels in the NF-κB signaling network.

Change to model	Biological interpretation	Simulated results
Decreased *c2a* (Equation 7)	Inhibition of induced IκBα protein due to heat shock proteins [39]	Figure 4A
Decreased [IkBaNFkB(0)] (Equation 11)	Reduction of steady state p65 protein levels due to Hsp72 overexpression (unknown mechanism)	Figure 5A
Decreased [IKKn(0)] (Equation 2)	Reduction of neutral IKK protein levels due to Hsp72 interactions with NEMO preventing proper IKK complex formation [15]	Figure S5A

Hsp72 interactions with the NF-κB signaling network are interpreted as constant perturbations to rates or initial species corresponding to the relevant biological reaction. Equation numbers reference the complete model equations shown in Figure 2, with altered parameters colored in red (inhibition) or blue (enhancement).

The basal levels of total IκBα play a key role in maintaining proper levels of NF-κB activation in resting cells [22]. Since no noticeable difference was observed in either NF-κB DNA binding activity or nuclear translocation between BV2 and Hsp72 cells prior to TNFα treatment (Figures 1B and S1), initial IκBα concentrations would not be expected to change without a corresponding change in total NF-κB concentration. To check whether this is the case, the total levels of p65 NF-κB in resting cells were measured using western blot. The amount of total p65 protein was reduced significantly compared to control BV2 cells (Figure 4B–4C).

The model was simulated again, this time assuming that the concentration of total p65 present in the cell was reduced to 70% of the nominal concentration, implemented by reducing the total amount of [IkBaNFkB(t)] provided as the initial condition to the model. Simulations predicted that reduction of the total pool of p65 changes the equilibrium levels of IκBα protein by a similar amount, consistent with the experimental measurements (Figure 4C).

These results show that Hsp72 reduces the amount of p65 protein in the cell by some mechanism, and suggest the hypothesis that the amount of total IκBα protein is coordinately reduced to maintain a constant equilibrium level of basal NF-κB activity in unstimulated cells.

### Model suggests a distinct contribution of reduced p65 expression to altered NF-κB signaling in Hsp72 overexpressing cells

**Figure 5:**
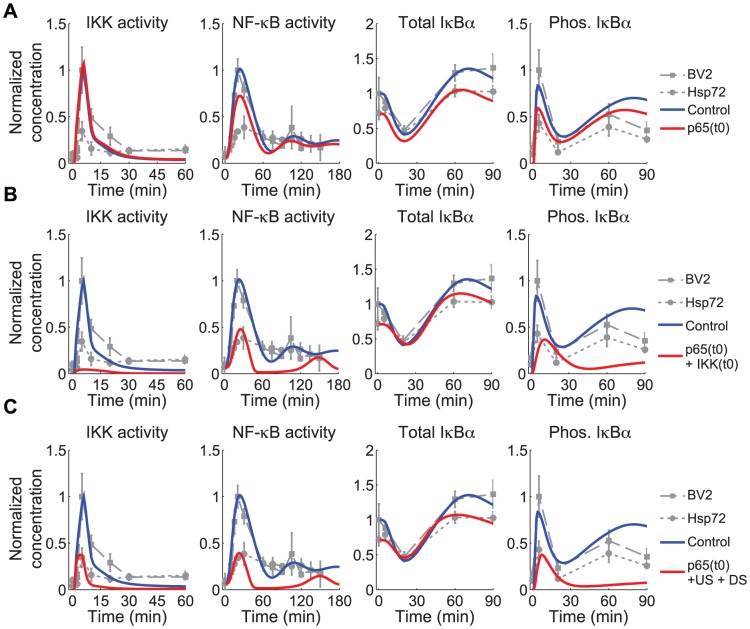
Model suggests plausible scenarios for Hsp72 regulation require both reduction of p65 and IKK inhibition. A. Dynamic simulations of model with p65 protein reduced to 70% (p65(t0)) show that reduction in total NF-κB partially accounts for the attenuated dynamics observed in Hsp72 expressing cells, but completely fails to account for reduction in IKK activity. B. Combining reduced p65 protein with a reduction in total IKK protein (to 5.5% of nominal levels) reduces peak IKK and NF-κB activation levels similar to experiments, suggesting the combined effect is one plausible scenario of interaction. C. The model with reduced p65 protein and interactions upstream (US, increasing inactivation rate *kiA20* 10-fold) and downstream (DS, simulated by decreasing IκBα phosphorylation rate, *kc2a*, by 6-fold) also match many qualitative features of the model. Gray lines are data renormalized such that basal IKK and NF-κB activity in BV2 cells is 10% of maximum.

In light of this result, how much of the observed attenuation of NF-κB activation can be attributed to a reduction in overall protein as opposed to the other potential Hsp72 interactions? Dynamic simulations of the model with reduced NF-κB protein levels, performed by decreasing [IkBaNFkB(0)] in the model to 70% of its nominal concentration, suggest that the effect of this is indeed reduced NF-κB activation in the absence of any further alterations in model parameters due to Hsp72 interactions (Figure 5A). However without regulating any additional points in the system, noticeably more IκBα is degraded at 20 min in simulations than what experimental evidence indicated, and peak amounts of phosphorylated IκBα only were reduced by a smaller amount than what experiments indicate. Furthermore, simulation indicates that the reduction in NF-κB levels will have a negligible effect on the amount of IKK activated, further supporting the earlier claim that at a minimum additional Hsp72 regulation at the level of IKK is required. Indeed, simulations of models lacking either assumed inhibition of IKK activation or reduction in total p65 fail to account for the observations from Hsp72 cells (Figure S4).

Given the unexpected decrease in p65 levels, one potential scenario that could account for the reduced IKK activity is that a similar reduction in the total amount of IKK present in the cell may also result from Hsp72 overexpression. The reduction in IKK simulated here could also be due to interactions of Hsp72 with NEMO that prevent proper IKK complex formation and reduce the pool of IKK able to be activated, similar to what was suggested by Ran et al. [15]. This was probed computationally, simulating the model with reduced p65 as before and additionally reducing basal IKK by modulating [IKKn(0)] (Figure 5B). The simulations suggest that under these conditions IKK activation can be reduced while also reducing peak NF-κB activation to a similar level as observed experimentally. When simulating the model this scenario was similar to others when assuming reduced p65 was accompanied by inhibition of IKK activation (Figure S5), suggesting that similar effects could be achieved using one or a combination of several mechanisms upstream of IKK. In all cases of reduced IKK activity in addition to decreased p65, however, the amount of IKK inhibition necessary to further attenuate NF-κB activation in simulation was much greater than observed experimentally (gray).

Similar results were observed when in addition to reducing p65, Hsp72 was assumed to inhibit both IKK activation and IκBα phosphorylation (Figures 5C and S5). With the additional downstream mechanism, the model was able to reduce peak NF-κB signaling sufficiently without requiring near complete inhibition of IKK activity as simulations suggest was necessary for significant reduction in NF-κB activation. Whether the additional downstream mechanism is essential, however, cannot be discerned with the present model.

In summary, the results of the computational probe of the possible Hsp72 regulatory scenarios support the hypothesis that two regulatory components are necessary: reduction in total p65 and inhibition of IKK. However due to the limited level of detail in the upstream model, it is not possible to distinguish whether Hsp72 is more likely to inhibit IKK activation by direct binding, to inhibit steps preceding IKK activation, or to reduce the amount of total IKK complex available in the cells. Additional downstream regulation at the level of IκBα phosphorylation appears plausible but not necessarily essential for NF-κB regulation in BV2 cells.

### Hsp72 overexpression associated with increased induction of IκBα mRNA

The primary function of NF-κB is its role as a transcription factor, directly or indirectly controlling the transcriptional activity of many important genes [24]. The expression of NF-κB target genes differs greatly in terms of their kinetics and can vary significantly depending on cell type [25]. Here we focused on the induction of two early gene targets whose expression is relatively well understood – A20 and IκBα – and which also play a critical role in the feedback regulation of the system. A20 and IκBα are both rapidly induced upon nuclear translocation and are believed to be under similar control [26], [27]. As such, one expects gene targets under similar control to be influenced similarly by Hsp72 attenuation of NF-κB activation.

**Figure 6:**
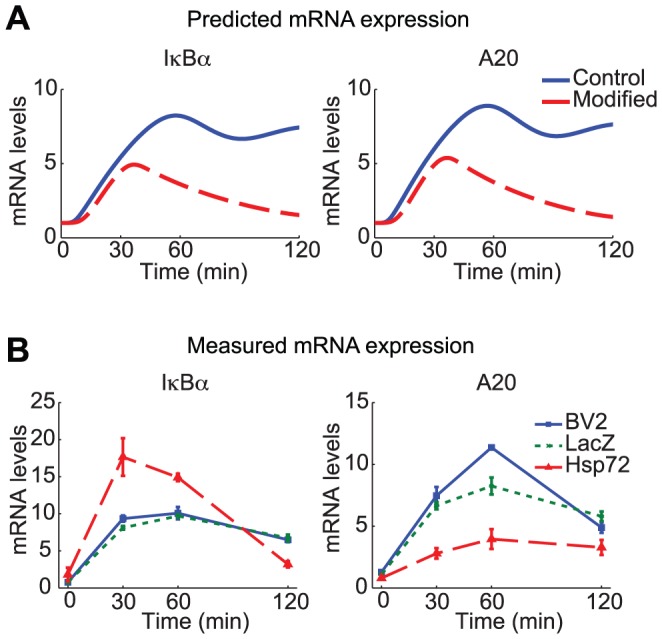
Hsp72 overexpression differentially regulates IκBα transcription. A. Model simulations with the nominal parameters predict that A20 and IκBα mRNA are similarly induced (blue solid lines). The model with modified parameters (*kiA20* set to 10-fold the nominal value, *kc2a* 1/6 the nominal value, and initial p65 70% of the nominal value) was simulated to find the transcript levels assuming that Hsp72 regulates the network by reducing total p65 and by inhibiting both upstream IKK activation and IκBα phosphorylation (red dashed lines). mRNA levels for IκBα and A20 transcripts are normalized with respect to their initial levels. B. IκBα and A20 mRNA transcript levels (fold change) in all three cell types were measured using qPCR at the indicated times following TNFα addition. IκBα and A20 transcripts are expressed similarly in BV2 and LacZ cells, while relative A20 is decreased in Hsp72 cells. Mean levels in Hsp72 cells are significantly different (P<0.05) from both BV2 and LacZ cells determined by ANOVA and Newman-Keuls *post hoc* tests at 30, 60 and 120 min (n = 3).

The model was used to compare the change in induced gene expression under the hypothesis that Hsp72 interacts at multiple points in the network. To simulate the response of a control BV2 cell, the model was simulated with the nominal parameter values and showed that IκBα and A20 transcripts are induced similarly, peaking 30–60 min and declining slightly by 120 min (Figure 6A). When the model is simulated assuming Hsp72 regulation of p65 levels, IKK activation, and IκBα phosphorylation (implemented assuming [NFkB(0)] is reduced to 70%, *kiA20* is increased by a factor of 10, and *kc2a* is reduced to 1/6), both mRNA expression levels are predicted to decrease significantly, again at similar levels owing to their identical transcriptional dependence on NF-κB and similar transcript stability.

To test this experimentally, mRNA transcript levels of both A20 and IκBα were measured in BV2, LacZ and Hsp72 cells. The relative induction of each was nearly identical in BV2 and LacZ cells and nearly matched the expected early activation profile (Figure 6B, blue). Furthermore in Hsp72 cells A20 expression was significantly decreased, with peak levels decreased by an amount that resembled simulations. Unexpectedly Hsp72 cells showed significantly increased relative expression of IκBα transcripts (Figure 6B, red). The discrepancy between induction of IκBα and A20 mRNA points to a mechanism by which Hsp72 overexpression differentially increases IκBα transcription despite less p65 activity.

We conclude from the above observations that Hsp72 overexpression affects mRNA transcript expression by increasing synthesis of IκBα mRNA following TNFα stimulation. This effect stands in contrast to A20 whose transcript levels are induced at lower levels as a consequence of reduced NF-κB activity, as expected from the model. At present the mechanisms underlying this observed effect are not known.

## Discussion

In this work we overexpressed Hsp72 in the microglial cell line BV2 to study its effects on NF-κB activation in response to the inflammatory cytokine TNFα. Our experiments demonstrated that Hsp72 overexpression reduced the amount of IKK kinase activity, the amount of NF-κB DNA binding activity, and the amount of phosphorylated IκBα protein. These results are largely consistent with other studies examining the anti-inflammatory effects of Hsp72 [14], [15], [16], [18], [28], [29].

We found that Hsp72 additionally reduced the steady state protein levels of both IκBα and p65. Computational analysis suggested that reductions in total IκBα were due to a shift in equilibrium induced by reductions in total NF-κB p65 protein rather than direct inhibition of IκBα synthesis itself. Model simulations of the response with the reduced basal protein levels also showed that this contributed in large part to attenuation in NF-κB activation, but that additional mechanisms are likely required. Specifically, modeling suggests that Hsp72 overexpression should act both by attenuating IKK activation rates and by reducing basal protein levels to regulate NF-κB activity in a manner consistent with our experimental observations in microglia. Whether downstream interactions are required could not be inferred from the model. However such a mechanism could permit finer control over inflammatory signaling by Hsp72 or provide a measure of redundancy to ensure more robust regulation. Our analysis therefore provides numerical evidence in support of the hypothesis that simultaneous activity of multiple modes of Hsp72 regulation are active in microglia, and is in agreement with other studies that suggested Hsp72 interacts at multiple points in the signaling pathway [16], [18].

It is important to keep in mind the limitations of the model when trying to infer conclusions about the biological system from its analysis. Mathematical models are, by necessity, approximate descriptions of the physical system that make a number of assumptions in order to translate physical reality into mathematical expressions. A key limitation of the present model is that it does not explicitly model Hsp72 interactions on the signaling network; rather it is assumed that Hsp72 overexpression effectively manipulates certain rate parameters or initial conditions of the model. Such an approach allowed us to find numerical evidence supporting broad hypotheses about where Hsp72 overexpression is expected to alter network dynamics. However, this assumed effect also limits our ability to examine particular and direct biochemical interactions, the explicit inclusion of which greatly increases the complexity of the model. Currently the kinetic data needed for this is unavailable. It is possible that detailed modeling of the explicit and dynamic interactions by Hsp72 in the future may yield valuable information.

Also of note is that in the model used here, the module describing kinetics of IKK activation is a coarse approximation of an intricate and deeply interconnected signaling network. While other studies have chosen to model this module in greater detail than in the model considered here (see, e.g. [27]), all share the critical structural assumption that the only feedback of downstream NF-κB signaling on IKK activation comes through induction of the A20 inhibitor. With only this feedback in place, it is structurally impossible for initial IKK activation to be altered by any Hsp72 interactions that occur downstream of IKK activity. Hence, the conclusion drawn here that Hsp72 regulation upstream of IKK is necessary in microglia is supported by alternative models of IKK activation sharing this hypothesis and is not restricted to the one employed in this study.

Our assertion that Hsp72 must inhibit IKK, based on our simulation results manipulating model parameters under the assumption that increased Hsp72 effectively decreases the rate of IKK activation, is not necessarily inconsistent with reports that have found that Hsp72 does not bind with IKK [14], [29]. In [14], reduced IκBα phosphorylation and NF-κB activation was observed in both transgenic mice and primary cultures of astrocytes and microglia overexpressing Hsp72. However, co-immunoprecipitation found that Hsp72 associates with NF-κB and IκB proteins, but not with NEMO/IKKγ. It is possible that while Hsp72 did not interact directly with IKK as suggested, it was still able to inhibit IKK activation by other means. In support of this, despite failing to see association between Hsp72 and IKK, Yoo et al. still observed a reduction in IKK activation in human respiratory epithelial cells [29]. A possible explanation accounting for this is that Hsp72 interacts upstream of IKK, either at the level of TRAF2 to prevent recruitment and activation of IKK, as suggested more recently by Dai et al. [16], or at the level of TRAF6 in the case of LPS signaling [19]. In this event IKK activation could be attenuated without necessitating direct association with the complex. Determining precisely whether Hsp72 acts directly at the level of IKK or with species involved in its activation further upstream could provide valuable information about potential stimulus-specific regulation. While interactions directly with IKK subunits are likely to inhibit any signaling that requires IKK activation, specific interactions with TRAF2 compared with TRAF6, for instance, could help tune the inflammatory response when subjected to multiple inputs acting through different receptor channels.

While our experiments demonstrate that basal p65 protein in untreated microglia decreases with Hsp72 overexpression (Figure 3), the mechanism by which Hsp72 overexpression reduces basal p65 is unclear. The promoter region of the *RELA* gene has three binding sites for SP-1 [30], pointing to regulation at the level of gene transcription. Regulation of NF-κB by microRNAs may offer another possible explanation. Increasing evidence points to extensive microRNA regulation of NF-κB signaling [31] and cerebral ischemia [32]. Furthermore, many known microRNAs are predicted to target *RELA* transcripts and other transcripts involved in the NF-κB pathway [33], [34].

Alternatively, regulation of other NF-κB isoforms as a result of Hsp72 overexpression could theoretically account for reduced total p65 protein. The p65 subunit dimerizes with other NF-κB subunits to form, for example, stable heterodimers with p50 and less stable p65 homodimers [35]. Conceivably reduction in available p50 could sequester less p65 in stable heterodimers and thereby reduce total p65 levels. Interestingly the *NFKB1* gene that encodes the precursor to the p50 subunit is itself a known transcriptional target of NF-κB activation in response to TNFα in at least certain cell types [36], [37]. However, our experiments show no evidence that basal NF-κB DNA binding activity is altered (Figure 1C), making this direct feedback loop an unlikely mechanism to explain reduced steady state p65. However there are findings that mice deficient in the p50 NF-κB subunit have reduced brain injury following focal cerebral ischemia [38]. The reduction in p65 in microglia is in contrast to a study in respiratory epithelial cells that detected no change in total p65 protein despite high levels of HSP in the cells [29]. These different observations may be explained in part by cell type differences or due to the use of heat shock and sodium arsenite to induce Hsp72 rather than transfection as done here. Understanding whether Hsp72 interaction has any effect on other NF-κB isoforms such as p50 could prove enlightening.

Our finding that induction of IκBα mRNA is increased in Hsp72 cells raises the questions what is the mechanism by which this is achieved, and what is its functional effect? The increase in IκBα mRNA induction is confounded by the other experimental observations from this study showing that total IκBα protein is expressed at reduced levels in the same cells while A20 mRNA expression simultaneously decreases. Model simulations under the assumed mechanisms of Hsp72 regulation predict that induction of A20 mRNA is decreased as expected owing to decreased NF-κB activity, but wrongly predict that IκBα mRNA expression should decrease as it is believed to be under similar transcriptional control by NF-κB. This is suggestive of some Hsp72-dependent means of regulation not yet identified. Evidence exists that heat shock (though not specifically Hsp72) can increase IκBα expression. Heat shock was reported to activate the human IκBα promoter in certain cell types [39]. However the model, simulated assuming that Hsp72 overexpression reduces expression of IκBα by reducing the rate *c2a*, predicts that induction of IκBα in the absence of other regulation dramatically alters NF-κB signaling in a way completely inconsistent with experiments (Figure 4A). This suggests that such a direct effect is highly unlikely under this assumed regulatory action. A more recent report by Dunsmore et al. also found that heat shock was able to upregulate IκBα expression, but concluded that it did so primarily by means of increasing transcript stability more so than by direct induction of the IκBα gene [40]. The mechanism was found to depend on p38 MAPK kinase, whose activation is also induced in response to TNFα and other inflammatory cytokines [41]. Therefore crosstalk between the two pathways, microRNA regulation, and how these are affected by Hsp72 might be fruitful areas of research.

A different possibility to explain differential induction of IκBα mRNA is that an alternative NF-κB subunit compensates for the reduced expression of p65 to induce IκBα rather than A20. The IκBα promoter is known to exhibit specificity to the different NF-κB subunits [42]. While the p50 and p50/RelB dimers have little effect on its activation and seem unlikely candidates, the c-Rel subunit is still able to activate IκBα transcription, albeit to a lesser degree than p65-containing dimers. Further experiments examining the expression and activation of c-Rel could address this.

Analysis of the model combined with experiments gave us ample evidence suggesting that regulation at the level of IKK is required, but did not permit us to pinpoint the exact mechanisms of Hsp72 regulation of IKK. For instance, the profile of IKK activation was predicted to change in terms of amplitude and timing based on the means of inhibition used (Figure S5). However, caution should be used in ruling out one mechanism over the other until the model is further validated. This is due partially to the large number of simplifying assumptions made in the signaling pathway from ligand-receptor binding to IKK activation. This pathway involves numerous signaling proteins and a rich network of post-transcriptional modifications. Additionally there are a number of regulators, in particular A20, which can inhibit or activate the response at multiple points [43]. Further characterization of this pathway and the kinetic interactions will help to better identify the steps involved in IKK activation and create models with better descriptive and predictive powers.

The mathematical model, while being useful for identifying discrepancies in qualitative behavior between mechanisms, was limited in its ability to reproduce certain features of NF-κB activation and IκBα profiles observed from Hsp72 cells. Structural constraints in the model limit its ability to generate a low amplitude, non-oscillating signal as the population average data suggests is present in Hsp72-expressing cells (Figure 1B, red) while also synthesizing and phosphorylating sufficient IκBα at later times. This may be in part due to the failure of population level measurements to accurately describe single cell NF-κB signaling. Single cell studies show that oscillatory behavior in individual cells may be masked by only considering bulk population averages [44], [45], [46], [47]. Keeping this in mind, comparisons between model simulations and experimental data were largely restricted to the initial period of activation during which cells stimulated with a high dose of TNFα tend to respond similarly before losing synchrony at later times [47]. Therefore the main conclusions presented in this paper suggesting that Hsp72 regulation must alter basal p65 protein levels and act to inhibit IKK activation during the first 30 minutes are expected to hold even when taking into account oscillations. An alternative approach using fluorescent reporting to track single cell trajectories of NF-κB activation may prove useful in developing a more accurate model to further study Hsp72 regulation of the network.

An aspect that is also important in interpreting results from our *in silico* analysis is the dependence of the model on rate parameters whose values are unknown. Many of the nominal parameter values were obtained in [21] by finding parameters that minimized the error between the model simulations and population level measurements of NF-κB signaling in BV2 cells. However given the limited amount of data used in identification and the large number of unknown parameters, the identified parameter set is not unique and hence many other sets could provide a similar response. While it is believed to be a general property of biological systems that the response exhibits sloppy parameter sensitivities and therefore leaves parameter values poorly constrained [48], we repeated the simulations when assuming that all model parameters – both rate constants and initial concentrations – are uncertain to see whether the results change with a different set of parameters. The results indicate some variability in the response depending on the sampling of the parameters, but that the average response when parameters are uniformly distributed in an interval of +/−20% of the fixed values is nearly identical to that using the individual parameter sets (Figures S6 and S7). Therefore, the results appear to be robust at least in the region of parameter space near the identified parameters. However for all high dimensional models with unknown parameters, including the one used here, the possibility still exists that other parameter sets far from those considered here could be plausible and potentially lead to different results, which must always be kept in mind.

**Figure 7:**
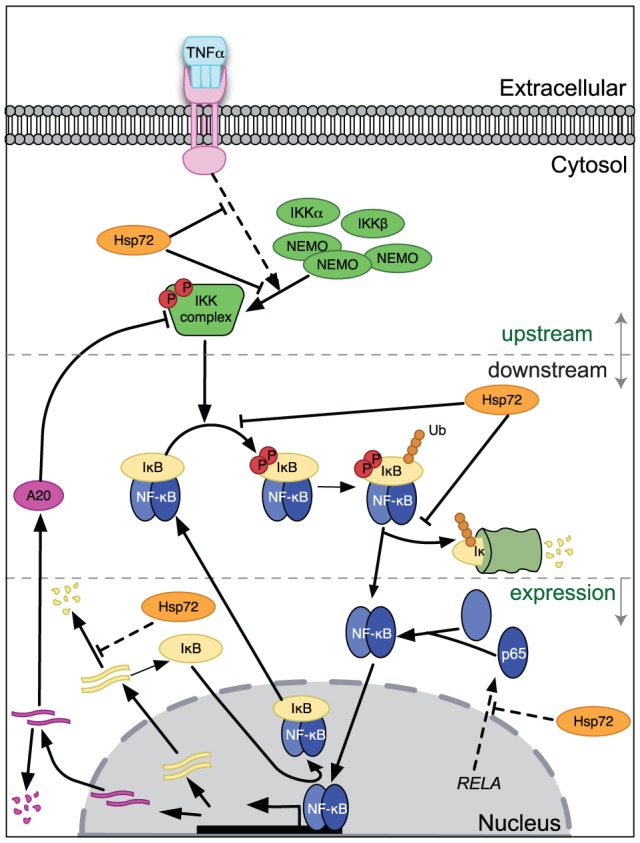
Updated mechanistic diagram for Hsp72 regulation of NF-κB signaling in microglia. Hsp72 regulation at the upstream level inhibiting IKK is an essential component in microglia, although the precise biomolecular mechanism cannot be discriminated. Hsp72 also plays a critical role in reducing synthesis of the p65 NF-κB subunit. Additionally, Hsp72 appears to increase the induction of IκBα transcript. Downstream of IKK, Hsp72 regulation may act to decrease phosphorylation and/or other steps in the stimulus-induced degradation of IκBα, though it appears less essential than the other two levels of regulation in this particular cell type and context.

This study used a combination of experimental observations and mathematical modeling to provide new insight into how Hsp72 regulates NF-κB activation in microglia (Figure 7). This study provided experimental evidence supported by model simulations that make a strong argument for the necessity for inhibition of IKK activation in microglia, which was an open question based on prior literature. Furthermore it uncovered a novel mechanism by which Hsp72 overexpression downregulates p65 protein, which has the effect of partially attenuating NF-κB activation and decreasing initial IκBα protein. Our observation that IκBα transcripts are upregulated whereas A20 transcripts are downregulated is still perplexing, and the mechanisms and consequences of this may prove interesting in the future.

## Materials and Methods

### Cell culture

BV2 cells, a mouse microglia cell line and kind gift from Dr. K. Andreasson at Stanford University, and Hsp72 and LacZ cell lines made by infecting BV2 cells with retrovirus LXSN-Hsp72 or -LacZ and neomycin selection, were used. Supernatants from Ψ2 packaging cells expressing LXSN/Hsp72 or LXSN/LacZ were used for infection. BV2 cells seeded in 24 well plates at 4×10^5^ cells/well were 60–70% confluent the following day and infected with packaging cell supernatant. Infected cells were stained for Hsp72. Cells from positive staining wells were subcloned and Western Blot confirmed expression. Subclones were stored in liquid nitrogen. Assays were run from the same clone and Hsp72 expression level was always confirmed by Western Blot before use in experiments. Cell lines were cultured in Dulbecco's Modified Eagle's medium (DMEM, GIBCO by Life Technologies, Carlsbad, CA) supplemented with 8% Fetal Bovine Serum (Hyclone, South Logan, UT), Penicillin (100 U/ml, GIBCO), and Streptomycin (100 µg/ml, GIBCO). Cells were passaged every four days and used between passages 10–20.

### Measurement of activated NF-κB p65

BV2, BV2-Hsp72 and BV2-LacZ cells were seeded at 4×10^5^ cells per well in six well plates 36 hrs prior to treatment with 10 ng/ml recombinant mouse TNFα (R&D Systems, Minneapolis, MN). Cells were then harvested for protein at the indicated times with Phosphosafe Extraction buffer (Novagen, Darmstadt, Germany) supplemented with 0.01 volume Protease Inhibitor cocktail (Sigma, St. Louis, MO) and 5 mM DTT before use. Protein concentration was measured using the Coomassie Plus assay (Pierce, Rockford, IL). 25 µg total protein/sample was transferred to a pre-chilled Eppendorf tube and brought to 25 µl with complete lysis buffer. Aliquots were stored at −80°C until use for activated NF-κB p65 measurement. Active NF-κB was measured using the Trans AM NFκB p65 Transcription Factor Assay Kit (Active Motif, Carlsbad, CA, cat#40096) according to the manufacturer's instructions on 20 ug total protein/sample. Three cultures were assayed for each group. Standards were prepared from recombinant p65 (Active Motif).

### Total and phospho-IκBα (Ser32) measurement

Total and phosphorylated IκBα were measured after TNFα treatment using sandwich ELISA kits from Cell Signaling (Danvers, MA, #7360 for total and #7355 for phospho). Cells seeded at 4×10^5^ cells/ml were treated on day 3 with 10 ng/ml TNFα. Cell lysates were prepared and 250 ug total protein, measured by BCA assay, was used for total or phospho-IκBα (Ser32) measurement, according to the kit instructions. Standard curves for phospho-IκBα (Ser32) measurement were made using phospho-IκBα control from Active MOTIF. Total and phospho-IκBα proteins were also run on Westerns to confirm activation.

### IKK measurement

IKK activity was measured by immunoprecipitation of IKK trimers, followed by a kinase assay/ELISA using a modification of the K-LISA IKK Inhibitor Screening Kit (Calbiochem, Billerica, MA, cat# CBA044). Cells were seeded and treated as above, protein was prepared following the kit instructions, and concentration determined by Coomassie Plus Assay. A total of 500 µg protein/sample was incubated at 4°C for 5 hrs with 5 µg goat anti-IKKγ antibody M18 (Santa Cruz Biotechnology, Dallas, TX, Cat# SC8256) with shaking, followed by overnight incubation with shaking with 50 µl 2× diluted Protein G-Sepharose (Sigma) previously washed in complete lysis buffer. Beads were then centrifuged for 5 min at 13,000 rpm 4°C, the post-immunoprecipitation supernatant removed, and beads were washed in the 1× kinase assay buffer from the K-LISA kit. Beads were then incubated with shaking in an incubator for 1 h at 30°C in 75 µl 1× kinase assay buffer containing 150 ng GST-IκBα and 1× ATP/MgCl_2_ mix from the kit. Beads were then centrifuged at 13,000 rpm for 5 min at 4°C, and 60 µl of supernatant was transferred to a well of the glutathione coated 96-well plate provided with the K-LISA kit. Two-fold serial dilutions of the recombinant IKKβ provided with the kit were run as standards, but omitting IKK inhibitor. In addition the post-immunoprecipitation supernatant was concentrated 20× and run to demonstrate that all IKK activity was depleted from the supernatant. In all cases this sample showed no IKK activity. The plate was incubated 30 min at 30°C to allow the GST-IκBα to bind, and subsequent processing was done according to the vendor's instructions. Final concentrations measured were normalized to the total amount of protein used in a given experiment.

### mRNA transcript measurement

Total RNA was isolated with TRIzol (Invitrogen, Carlsbad, CA, USA) and reverse transcription performed using the TaqMan MicroRNA reverse Transcription Kit (Applied Biosystems, Carlsbad, CA, USA) on equal amounts of total RNA (600 ng) using 100 mM dNTPs, 75 U reverse transcriptase, 10 U RNase inhibitor, and specific mRNA reverse transcriptase primers (Applied Biosystems) at 25°C for 10 min, 37°C for 120 min, and 85°C for 5 min. PCR reactions used the TaqMan MicroRNA Assay Kit (Applied Biosystems) at 95°C for 10 min, followed by 40 cycles of 95°C for 15 seconds and 60°C for 1 min. Each reaction contained 0.75 µl of the RT reaction product, 5 µl TaqMan 2×Universal PCR Master Mix (Applied Biosystems) in a total volume of 10 µl using the 7900HT (Applied Biosystems). Predesigned primer/probes for mRNAs and mouse GAPDH were from Applied Biosystems. The expression of mRNAs was normalized using GAPDH as the internal control. Measurements were normalized to GAPDH (ΔCt) and comparisons calculated as the inverse log of ΔΔCT to give the relative fold change for all mRNA levels. The PCR experiments were repeated 3 times, each using separate sets of samples.

### p65 staining

Cells plated at 4×10^5^/well were harvested 0, 20 and 40 min after addition of 10 ng/ml TNFα the following day. Cells were washed once with 1× PBS followed by fixation for 20 min in 4% paraformaldehyde then 10 min in 0.3% H_2_O_2_ for permeabilization. Primary rabbit anti-p65 antibody (Abcam, Cambridge, MA, Cal# is 16502) was applied at 1∶800 dilution, cells were then incubated at 4°C overnight. Alexa Fluor 488 conjugated goat anti-rabbit secondary antibody (Invitrogen, Cal# is A11008) was then applied for 1 hr. Five pictures were taken from each well for analysis. To quantify the images, the fluorescence intensity of the nuclei and the fluorescence intensity of the whole cells were measured, and the proportion of total staining that was nuclear was determined.

### Western blot

To assess Hsp72 overexpression equal amounts (25 µg) of protein were separated on a polyacrylamide gel (Invitrogen), and electrotransferred to Immobilon polyvinylidene fluoride membrane (Millipore Corp., Billerica, MA). Membranes were blocked and incubated overnight with primary antibody against Hsp72 (1∶1000, #SMC100A, StressMarq, Victoria, BC, Canada, cat# SMC100A) and β-actin (1∶1000 LiCOR Bioscience, Lincoln, NE, cat# 926-42210), washed and incubated with 1∶15000 anti-rabbit antibody (926-32221, LiCOR Bioscience) and anti-mouse antibody (926-32220, LiCOR Bioscience). Immunoreactive bands were visualized using the LICOR Odyssey infrared imaging system according to the manufacturer's protocol. Densitometric analysis was performed using ImageJ software (NIH). Band intensities were normalized to β-actin.

For p65 protein levels, 35 ug and 70 ug of total protein were assayed for each sample. After separation and transfer, membranes were blocked and incubated overnight with primary antibody against NF-κB p65 (1∶200, Santa Cruz, cat# sc-109,) and β-actin (1∶5000, Sigma, cat# A1978), washed and incubated with 1∶15000 anti-rabbit antibody (926-32221, LiCOR Bioscience) and anti-mouse antibody (926-32220, LiCOR Bioscience). Immunoreactive bands were visualized and analyzed as above.

For total and phosphor IκBα, protein samples were prepared following the IκBα ELISA kit instructions, Cell Signaling (#7360 for total and #7355 for phospho). 50 ug total protein was used for each sample. After protein separation and membrane transfer, membranes were blocked and incubated overnight with primary antibody against either total IκBα (1∶1000, Cell Signaling, cat# 9242) or phospho-IκBα (Ser32/36,1∶500, Cell Signaling, cat# 9246) and β-actin (1∶1000, LiCOR Bioscience, cat# 926-42210), washed and incubated with anti-rabbit antibody (1∶15000, LiCOR Bioscience, cat# 926-32221) and anti-mouse antibody (1∶15000, LiCOR Bioscience, cat# 926-32220). Immunoreactive bands were visualized and analyzed as above.

### Statistical analysis

Experimental data were analyzed using one-way analysis of variance (ANOVA) to detect significant differences in mean values among cell types. Data sets for which a significant effect was determined were further analyzed using the Newman-Keuls multiple comparison *post hoc* test. For time course data, each time point was considered independently of the others since independent cell populations were harvested. Total p65 levels quantified from western blot were compared using one-sample Student's t-test with the null hypothesis that the test group had mean unity since each group of cells was first normalized by the quantity measured in BV2 control cells. Results were considered significantly different at confidence level P<0.05. Experimental data shown is mean +/− SD.

### Mathematical modeling

The deterministic ordinary differential model from [21] was modified slightly to permit constitutive degradation of phosphorylated intermediates of IκBα and allow nuclear import of the IκBα:NF-κB complex. Parameters for degradation and synthesis were adjusted to fit rates suggested in [22] to more closely match the additional experimental time courses measured in this study. Numerical simulations were performed using custom code written in Matlab R2010b (MathWorks, Natick, MA). Briefly, conserved protein quantities of total NF-κB and IKK were initialized to assumed concentrations and the system was simulated without stimulus until all remaining species reached equilibrium. Simulations with stimulus began from these equilibrium concentrations at 0 min but with stimulus set to present. Simulations of potential Hsp72 regulation scenarios altered kinetic rates and/or initial concentrations prior to equilibration, and simulations were performed with these modified parameters. All model reactions and initial conditions are provided in the Supplement Tables S1, S2. Matlab source code is available for download at http://www.bsse.ethz.ch/ctsb/tools/microglia_nfkb_model.

## Supporting Information

Figure S1 **Immunostaining of NF-κB p65 nuclear translocation.** A. BV2, LacZ, and Hsp72 cells were fixed following TNFα treatment at the time points indicated and stained for p65. Representative images are shown. Nuclear translocation of p65 is significantly higher 20 min following stimulus in all three cell types, but the proportion of nuclear p65 is decreased in Hsp72 cells compared to control cells. B. Quantification of immunostaining results plotting the fraction of fluorescence staining in the nucleus to the total cellular fluorescence.
(TIF)

Figure S2 **Additional simulations of upstream Hsp72 interactions.** Results of simulated Hsp72 interactions occurring upstream at the level of IKK activation showing the response of the species shown in the column heading. A. Inhibition of TNF-induced activation (*ka*). B. Inhibition of recycling rate from inactivated form unable to be stimulated to neutral form capable of activation (*kp*). C. Enhancement of auto-phosphorylation inactivation (*ki*). D. Enhancement of inactivation rate which is dependent on A20 regulation (*kiA20*).
(EPS)

Figure S3 **Simulations of additional downstream Hsp72 interactions.** Results of simulated Hsp72 interactions occurring downstream at the level of the IκBα:NF-κB complex showing the response of the species shown in the column heading. A. Inhibition of phosphorylation by IKK (*kc2a*). B. Inhibition of proteasomal degradation (*kupd*). C. Inhibition of E3-ligase recruitment to phosphorylated IκBα (*kua1*). D. Inhibition of poly-ubiquitination (*kuc1*).
(EPS)

Figure S4 **Simulations of additional implausible scenarios of Hsp72 regulation.** Simulation results showing scenarios of Hsp72 regulation that are inadequate to account for key experimental observations in addition to those shown in the main text. A. Reduction of only total IKK concentration by the factor shown at right cannot reduce amount of basal total IκBα. B. Reduction of initial IKK by 1/8 and downstream (DS) inhibition of IκBα phosphorylation rate (*kc2a* set to 1/5 nominal value) cannot reduce basal IκBα. C. Reduction of total p65 and only inhibition of DS signaling component (*kc2a* set to 1/10 nominal value) is not able to reduce IKK activation.
(EPS)

Figure S5 **Simulations of additional plausible scenarios of Hsp72 regulation.** Simulation results showing scenarios of Hsp72 regulation that include the necessary components and may be plausible. Models that include total p65 reduced by 70% plus the additional mechanisms specified. (A–D) DS denotes inhibition of the IκBα phosphorylation rate (*kc2a*).
(EPS)

Figure S6 **Simulation results consistent in presence of parameter uncertainty.** Simulations in the main text were repeated assuming that model parameters and initial conditions are uncertain, distributed uniformly in an interval +/−20% centered around the mean. Simulations were performed using 100 randomly sampled parameter sets and averaged for comparison. Thin lines indicate results from random samples, while thicker lines show the average response. Blue indicates parameters centered around the nominal set used to model control cells; red indicates parameters centered around the modified parameters assumed to be altered by Hsp72. A. Modified parameter *ka* to 1/16 of the nominal value; compare with Figure 3A. B. Modified parameter *kc2a* to 1/32 of the nominal value; compare with Figure 3B. C. Modified parameter *c2a* to 1/30 the nominal value. Compare to Figure 4A.
(TIF)

Figure S7 **Simulation results consistent in presence of parameter uncertainty.** Simulations in the main text were repeated assuming that model parameters and initial conditions are uncertain, distributed uniformly in an interval +/−20% centered around the mean. Simulations were performed using 100 randomly sampled parameter sets and averaged for comparison. Thin lines indicate results from random samples, while thicker lines show the average response. Blue indicates parameters centered around the nominal set used to model control cells; red indicates parameters centered around the modified parameters assumed to be altered by Hsp72. A. Modified initial condition [IkBaNFkB(0)] to 70% of the nominal value; compare with Figure 5A. B. Modified initial conditions [IKKn(0)] to 1/18 of the nominal value and [IkBaNFkB(0)] to 70% of the nominal value; compare with Figure 55. C. Modified initial condition [IkBaNFkB(0)] to 70% of the nominal value, parameter *kc2a* to 1/6 the nominal value, and parameter *kiA20* to 10-fold the nominal value; compare with Figure 5C.
(TIF)

Table S1 **Initial conditions for simulations.** All other species in the model were assumed to have zero concentration and the model was simulated until equilibrium was reached. Stimulus was then added following the equilibration period.
(DOC)

Table S2 **Reactions and parameters for downstream model describing NF-κB signaling pathway.**
(DOC)
