# Supplementary figures and images for: Overexpression of Heat Shock Protein 72 Attenuates NF-κB Activation Using a Combination of Regulatory Mechanisms in Microglia
Source: PLoS Comput Biol. 2014 Feb 6;10(2):e1003471. doi: 10.1371/journal.pcbi.1003471 (PMC3916226; doi:10.1371/journal.pcbi.1003471)

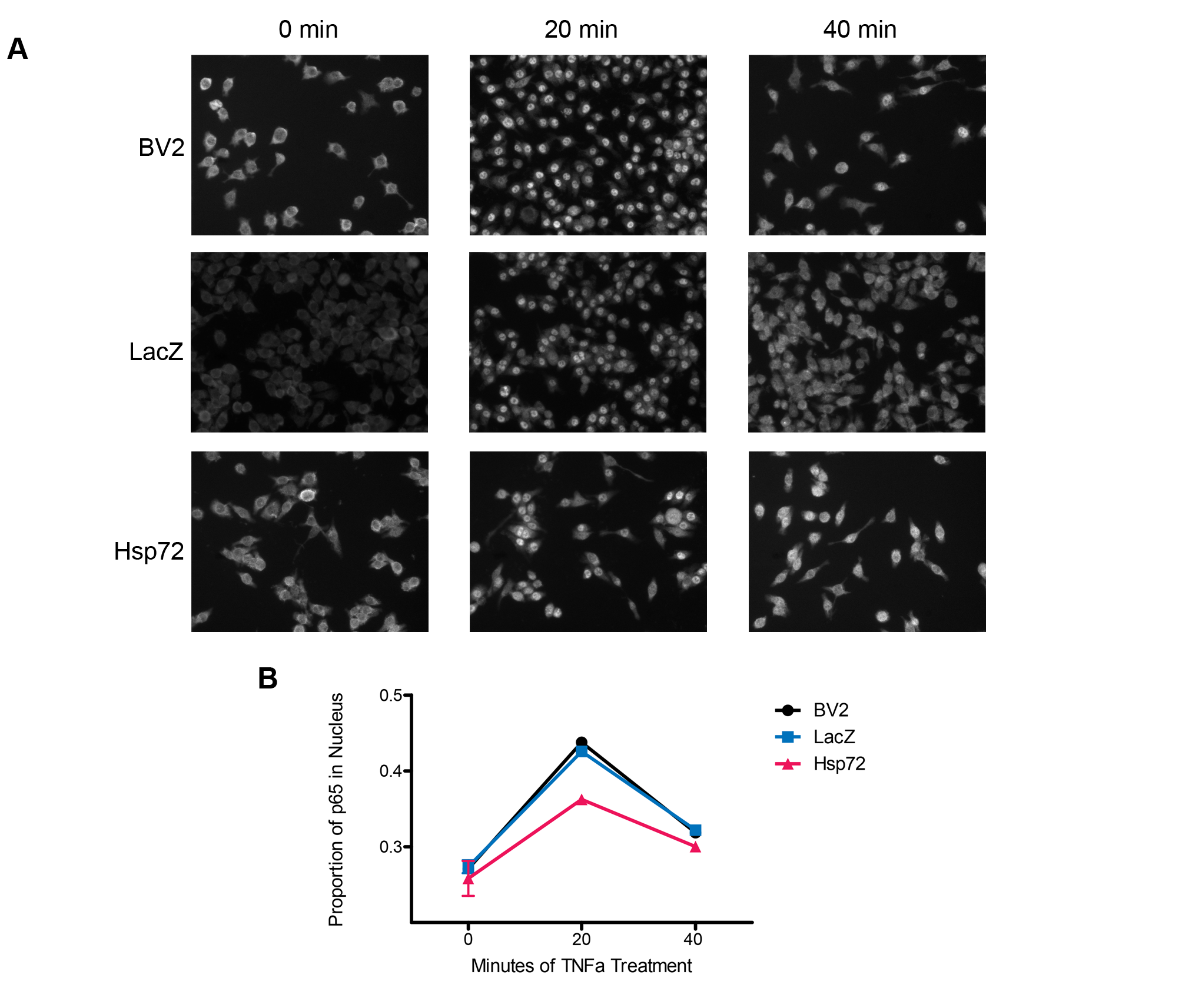

Supplement: Figure S1 — Immunostaining of NF-κB p65 nuclear translocation. A. BV2, LacZ, and Hsp72 cells were fixed following TNFα treatment at the time points indicated and stained for p65. Representative images are shown. Nuclear translocation of p65 is significantly higher 20 min following stimulus in all three cell types, but the proportion of nuclear p65 is decreased in Hsp72 cells compared to control cells. B. Quantification of immunostaining results plotting the fraction of fluorescence staining in the nucleus to the total cellular fluorescence. (TIF) [file pcbi.1003471.s001.tif]

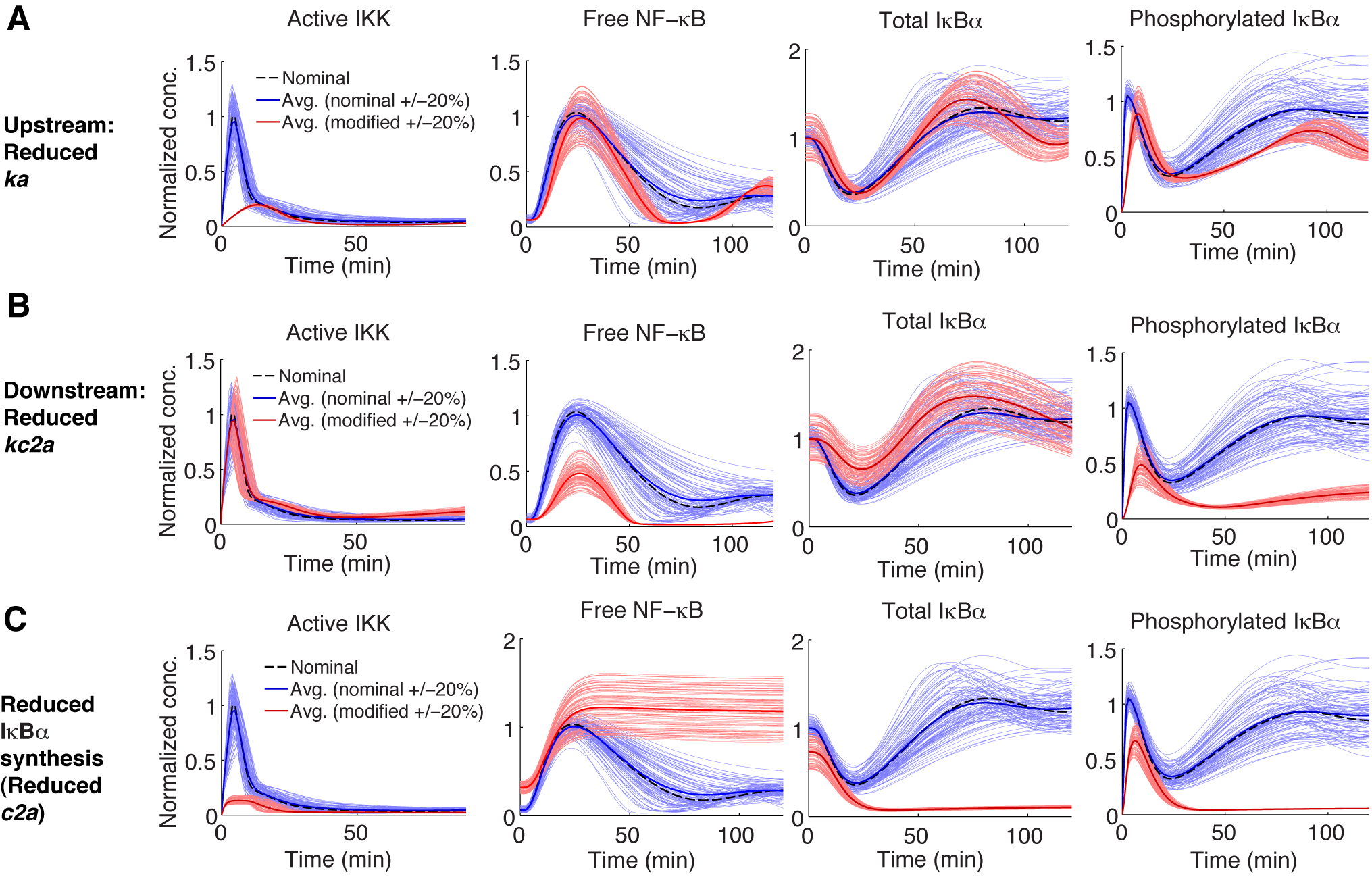

Supplement: Figure S6 — Simulation results consistent in presence of parameter uncertainty. Simulations in the main text were repeated assuming that model parameters and initial conditions are uncertain, distributed uniformly in an interval +/−20% centered around the mean. Simulations were performed using 100 randomly sampled parameter sets and averaged for comparison. Thin lines indicate results from random samples, while thicker lines show the average response. Blue indicates parameters centered around the nominal set used to model control cells; red indicates parameters centered around the modified parameters assumed to be altered by Hsp72. A. Modified parameter ka to 1/16 of the nominal value; compare with Figure 3A. B. Modified parameter kc2a to 1/32 of the nominal value; compare with Figure 3B. C. Modified parameter c2a to 1/30 the nominal value. Compare to Figure 4A. (TIF) [file pcbi.1003471.s006.tif]

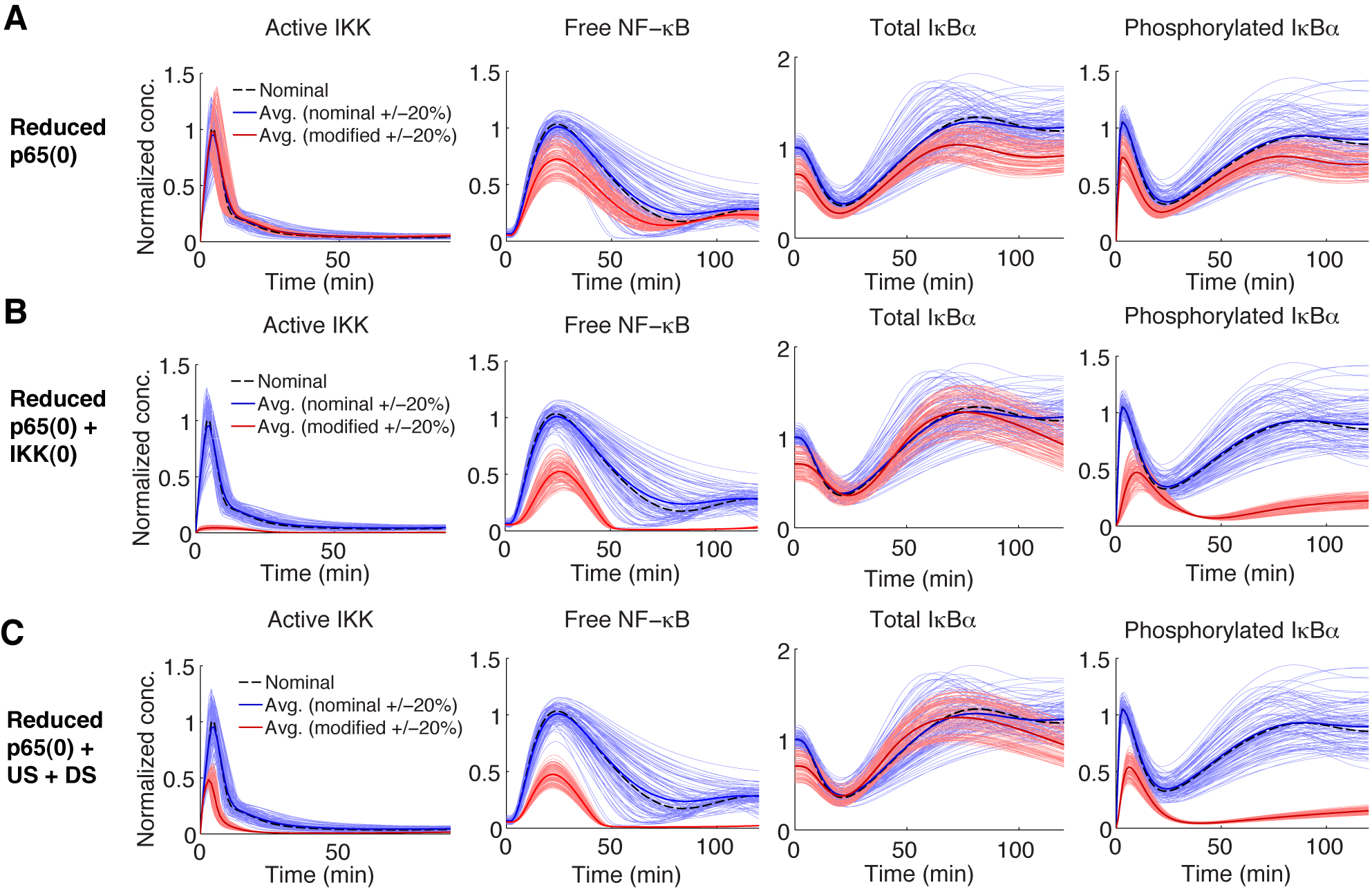

Supplement: Figure S7 — Simulation results consistent in presence of parameter uncertainty. Simulations in the main text were repeated assuming that model parameters and initial conditions are uncertain, distributed uniformly in an interval +/−20% centered around the mean. Simulations were performed using 100 randomly sampled parameter sets and averaged for comparison. Thin lines indicate results from random samples, while thicker lines show the average response. Blue indicates parameters centered around the nominal set used to model control cells; red indicates parameters centered around the modified parameters assumed to be altered by Hsp72. A. Modified initial condition [IkBaNFkB(0)] to 70% of the nominal value; compare with Figure 5A. B. Modified initial conditions [IKKn(0)] to 1/18 of the nominal value and [IkBaNFkB(0)] to 70% of the nominal value; compare with Figure 55. C. Modified initial condition [IkBaNFkB(0)] to 70% of the nominal value, parameter kc2a to 1/6 the nominal value, and parameter kiA20 to 10-fold the nominal value; compare with Figure 5C. (TIF) [file pcbi.1003471.s007.tif]
