# Supplementary material for: Overexpression of Heat Shock Protein 72 Attenuates NF-κB Activation Using a Combination of Regulatory Mechanisms in Microglia
Source: PLoS Comput Biol. 2014 Feb 6;10(2):e1003471. doi: 10.1371/journal.pcbi.1003471 (PMC3916226; doi:10.1371/journal.pcbi.1003471)
Supplement: Table S1 — Initial conditions for simulations. All other species in the model were assumed to have zero concentration and the model was simulated until equilibrium was reached. Stimulus was then added following the equilibration period. (DOC) [file pcbi.1003471.s008.doc]

Table S1: Initial conditions for simulations. All other species in the model were assumed to have zero concentration and the model was simulated until equilibrium was reached. Stimulus was then added following the equilibration period.

| **Species** | **Symbol** | **Initial value** |
| --- | --- | --- |
| Free NF-κB protein (cytoplasm) | NFkB | 0.080 μM in control cells  0.056 μM (assume 70% when Hsp72 present from Figure 3C) |
| Native IKK complex | IKKn | 0.08 μM |
| TNFα stimulus | TNF | 0, no stimulus present  1, TNFα stimulus present |
