# Supplementary material for: Overexpression of Heat Shock Protein 72 Attenuates NF-κB Activation Using a Combination of Regulatory Mechanisms in Microglia
Source: PLoS Comput Biol. 2014 Feb 6;10(2):e1003471. doi: 10.1371/journal.pcbi.1003471 (PMC3916226; doi:10.1371/journal.pcbi.1003471)
Supplement: Table S2 — Reactions and parameters for downstream model describing NF-κB signaling pathway. (DOC) [file pcbi.1003471.s009.doc]

Table S2: Reactions and parameters for downstream model describing NF-κB signaling pathway

| **Reaction or constant** | **Rate** | | **Value** | **Notes and references** |
| --- | --- | --- | --- | --- |
| Cytoplasm/nucleus volume ratio | *kv* | | 5.0 | [1] |
| IKKn → IKKa (TNFα induced) | TNF**ka**[IKKn]*^ha^* / ([IKKn]*^ha^* + *kmma^ha^*) * *ka20*/(*ka20*+TNF*[A20]) | *ka* = 3.37 ×10^-4^ M ^-1^ s^-1^  *kmma* = 7.71 ×10^-3^ M  *ha* = 3.78  *ka20* = 8.26 ×10^-3^ M | | [1] |
| IKKa → IKKi (constitutive) | *ki**[IKKa]*^hi^* / ([IKKa]*^hi^* + *kmmi^hi^*) | *ki* = 9.23 ×10^-5^ s^-1^  *kmmi* = 1.27 ×10^-2^ M  *hi* = 9 | | [1] |
| IKKi → IKKn | *kp**[IKKi] | *kp* = 2.90 ×10^-4^ s^-1^ | | [1] |
| IKKa → IKKi (A20 feedback) | TNF**kiA20**[IKKa]* [A20] | *kiA20* = 6.39 ×10^-1^ s^-1^ | | [1] |
| NFkBn →NFkBn + A20t | *c1** [NFkBn]*^h^* /(*k*^2^ + [NFkBn]*^h^*) | *c1*=2.55 ×10^-7^ M^-1^ s^-1^  *k* = 0.065 μM  *h* = 2 | | [1] |
| A20t → A20t + A20 | *c2* | 3.2×10^-1^ s^-1^ | | Assumed same for A20 and IkBa |
| A20t→ | *c3* | 2.90 ×10^-4^ s^-1^ | | [1] |
| A20 → | *c4* | 6.0 ×10^-3^ s^-1^ | | [1] |
| IkBa + NFkB →IkBaNFkB | *ka1a* (forward) | | 1.0 ×10^0^ μM^-1^ s^-1^ | [1] |
| IkBaNFkB →IkBa + NFkB | *kd1a* (reverse) | | 5.0 ×10^-4^ s^-1^ | [1] |
| IkBan + NFkBn → IkBaNFkBn | *ka1a* (forward) | | 1.0 ×10^0^ μM^-1^ s^-1^ | [1] |
| IkBaNFkBn → IkBan + NFkBn | *kd1a* (reverse) | | 5.0 ×10^-4^ s^-1^ | [1] |
| NFkB → NFkBn | *ki1* (import) | | 1.58 ×10^-2^ s^-1^ | [1] |
| NFkBn → NFkB | *ke1* (export) | | 3.16 ×10^-4^ s^-1^ | [1] |
| IkBa →IkBan | *ki3a* (import) | | 1.32 ×10^-3^ s^-1^ | [1] |
| IkBan →IkBa | *ke3a* (export) | | 0.66 ×10^-3^ s^-1^ | [1] |
| IkBaNFkBn → IkBaNFkB | *ke2a* | | 1.0 ×10^-2^ s^-1^ | [1] |
| IkBaNFkB → IkBaNFkBn | *ki2a* | | 4.6 ×10^-3^ s^-1^ | [2] |
| NFkBn →NFkBn + IkBat | *c1a* * [NFkBn]^h^ /(k^h^ + [NFkBn]*^h^*) | | *c1a*=2.55 ×10^-7^ μM^-1^ s^-1^  *k* = 0.065 μM  *h* = 2 | [1] |
| tIkBa → tIkBa + IkBa | *c2a* | | 3.2 ×10^-1^ s^-1^ | Estimated; constrained 1-5 ×10^-1^ s^-1^ [3] |
| tIkBa → | *c3a* | | 2.52 ×10^-4^ s^-1^ | [1] |
| IkBa → | *c4a* | | 1 ×10^-3^ s^-1^ | Increased to be approximately 5 min half life [4] |
| NFkBIkBa → NFkB | *c5a* | | 2.2 ×10^-5^ s^-1^ | [1] |
| IKKa + IkBa → IKKa + pIkBa | *kc1a* | | 1.73 ×10^0^ μM^-1^ s^-1^ | [1] |
| IKKa + IkBaNFkB → IKKa + pIkBaNFkB | *kc2a* | | 1.73 ×10^0^ μM^-1^ s^-1^ | [1] |
| pIkBa → E3pIkBa | *kua1* | | 3.65 ×10^-3^ s^-1^ | [1] |
| pIkBaNFkB → E3pIkBaNFkB | *kua1* | | 3.65 ×10^-3^ s^-1^ | [1] |
| E3pIkBa → uIkBa | *kuc1* | | 3.65 ×10^-3^ s^-1^ | [1] |
| E3pIKbaNFkB → uIkBaNFkB | *kuc1* | | 3.65 ×10^-3^ s^-1^ | [1] |
| uIkBa → (induced) | *kupd* | | 5.0 ×10^-3^ s^-1^ | [1] |
| uIkBaNFkB → NFkB | *kupd* | | 5.0 ×10^-3^ s^-1^ | [1] |
| pIkBa → | *c4a* | | 1 ×10^-3^ s^-1^ | Assumed phosphorylated form still subject to constitutive degradation |
| pIkBaNFkB → NFkB | *c5a* | | 2.2 ×10^-5^ s^-1^ | See above |
| E3pIkBa → | *c4a* | | 1 ×10^-3^ s^-1^ | See above |
| E3pIKbaNFkB → NFkB | *c5a* | | 2.2 ×10^-5^ s^-1^ | See above |
| uIkBa → | *c4a* | | 1 ×10^-3^ s^-1^ | See above |
| uIkBaNFkB → NFkB | *c5a* | | 2.2 ×10^-5^ s^-1^ | See above |

**SUPPORTING INFORMATION REFERENCES**

1. Sheppard P, Sun X, Emery J, Giffard R, Khammash M (2011) Quantitative characterization and analysis of the dynamic NF-kappaB response in microglia. BMC Bioinformatics 12: 276.

2. Werner SL, Barken D, Hoffmann A (2005) Stimulus Specificity of Gene Expression Programs Determined by Temporal Control of IKK Activity. Science 309: 1857--1861.

3. Lipniacki T, Paszek P, Brasier AR, Luxon B, Kimmel M (2004) Mathematical model of NF-κB regulatory module. J Theor Biol 228: 195--215.

4. O'Dea EL, Barken D, Peralta RQ, Tran KT, Werner SL, et al. (2007) A homeostatic model of IκB metabolism to control constitutive NF-κB activity. Mol Syst Biol 3: 111.
